# Systematic evaluation of a ^171^Yb optical clock by synchronous comparison between two lattice systems

**DOI:** 10.1038/s41598-018-26365-w

**Published:** 2018-05-22

**Authors:** Qi Gao, Min Zhou, Chengyin Han, Shangyan Li, Shuang Zhang, Yuan Yao, Bo Li, Hao Qiao, Di Ai, Ge Lou, Mengya Zhang, Yanyi Jiang, Zhiyi Bi, Longsheng Ma, Xinye Xu

**Affiliations:** State Key Laboratory of Precision Spectroscopy, East China Normal University, Shanghai, 200062 China

## Abstract

Optical clocks are the most precise measurement devices. Here we experimentally characterize one such clock based on the ^1^*S*_0_-^3^*P*_0_ transition of neutral ^171^Yb atoms confined in an optical lattice. Given that the systematic evaluation using an interleaved stabilization scheme is unable to avoid noise from the clock laser, synchronous comparisons against a second ^171^Yb lattice system were implemented to accelerate the evaluation. The fractional instability of one clock falls below 4 × 10^**−**17^ after an averaging over a time of 5,000 seconds. The systematic frequency shifts were corrected with a total uncertainty of 1.7 × 10^**−**16^. The lattice polarizability shift currently contributes the largest source. This work paves the way to measuring the absolute clock transition frequency relative to the primary Cs standard or against the International System of Units (SI) second.

## Introduction

The past decade has seen rapid breakthroughs in the development of atomic clocks based on optical transitions, specifically the optical clocks^1,2^. State-of-the-art optical clocks^3–8^, achieving fractional frequency instabilities near 10^−16^ in a 1-s averaging time and systematic uncertainties of a few parts in 10^−18^, now surpass their microwave counterparts realizing the definition of the SI second in both aspects by two orders of magnitude^9,10^. The increasingly high measurement precision has led to new tests of fundamental physics, such as the gravitational redshift, and the constancy of fundamental constants^11–14^. Along with the verification of physical theories, dedicated optical networks for linking distant optical clocks are being established^15^, thereby enabling applications in fields including time and frequency dissemination, geodesy, and satellite-based navigation^16–19^. Further, these achievements will open up the possibility of a redefinition of the SI second in the future^20,21^.

To date, two different types of optical clocks are being developed worldwide^1,2^, specifically, the ion clock that uses a single ion stored in a radio-frequency trap, and the lattice clock that employs ensembles of neutral atoms confined in an optical lattice. For the lattice clock realization, atomic ytterbium with a spin-1/2 system, i.e., ^171^Yb, is recognized as one of the most promising candidates. Its so-called clock transition ^1^*S*_0_-^3^*P*_0_ was endorsed by the International Committee for Weights and Measures (CIPM) as a secondary representation of the second (i.e., one of the candidates for redefining the SI second)^22^. To the best of our knowledge, five laboratories so far have independently measured the absolute frequency, either by tracing to the primary Cs standards or deducing from the measured optical frequency ratios^23–27^. Among the above-mentioned measurements, systematic uncertainties of the clock transition in ^171^Yb are reported at levels 10^−16^–10^−17^. Because of a lack of auxiliary accurate optical-frequency references, some evaluations have to be performed using the self-comparison method. In this case, the stabilization of the clock laser on the atomic resonance involves two sequences that are interleaved in time, with the parameter alternating during the interrogation periods. The resulting frequency difference is regarded as the beat note of two optical clocks, with which the parameter dependence can be identified. However, comparisons of two independent lattice clocks have shown that the frequency instability is essentially limited by the clock laser and the Dick effect^6,28^. To accelerate the systematic uncertainty evaluation, synchronous interrogation of two lattice systems is preferable^29^. It is envisaged that the common-mode noise of the clock laser can be rejected and the atomic response to external fields can be fully characterized.

We have previously investigated the clock-transition spectrum in ^171^Yb^30^. In this paper, we further report on the systematic evaluation of our ^171^Yb optical lattice clock, which lays the groundwork for future absolute frequency measurements. Instead of using the conventional self-comparison method, two independent lattice systems are interrogated synchronously by an optical local oscillator with a fractional frequency instability of 1.3 × 10^−15^ at 1 s averaging time. The comparison allows us to achieve better clock stability, which helps uncover quickly the frequency shift sources that affect the clock accuracy.

## Results

### Experimental setup

**Figure 1:**
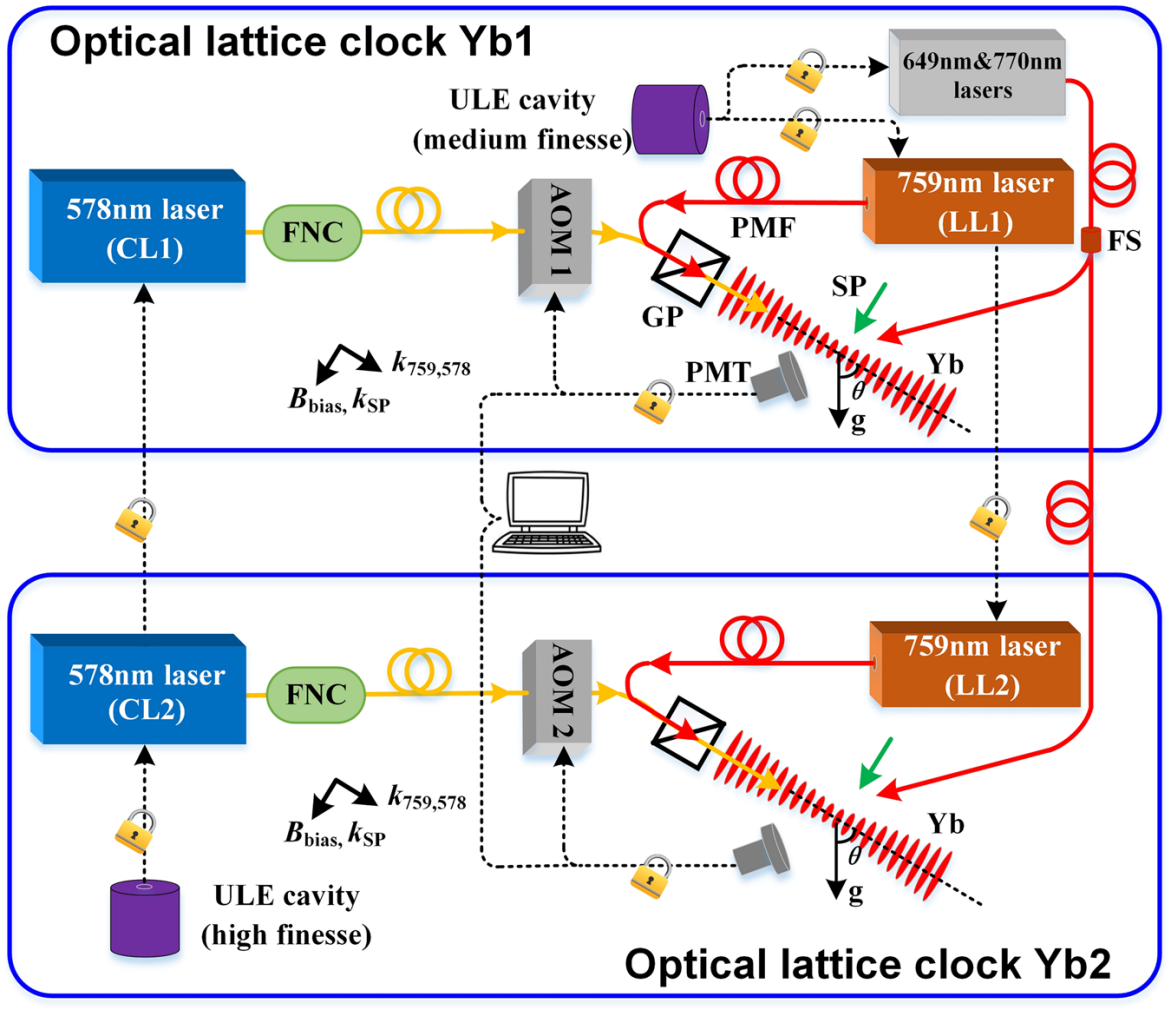
Schematic of the experimental setup. Two samples of cold ytterbium atoms in Yb1 and Yb2 are stored in optical lattices formed by 759-nm lasers LL1 and LL2, respectively. Both lattices are tilted at angles of $\theta$ = 54.8° with respect to gravity. In the presence of a bias magnetic field ${B}_{{\rm{bias}}}$, the population redistribution of Zeeman sublevels in the clock ground state is performed with 556-nm spin-polarized (SP) lasers. The 578-nm clock laser CL1 is phase locked to the cavity-stabilized laser CL2. Cold atoms are independently probed by two laser beams from CL1 and CL2. The respective clock-transition spectra, measured from the laser-induced fluorescence by photomultiplier tubes (PMTs), are normalized using two repumpers at 649 nm and 770 nm. Frequency corrections deduced from the excitation fractions are actively fed back to two separate acousto-optic frequency modulators (AOM1, AOM2), thus the clock laser frequencies are forced to align with the atomic resonance. A personal computer records the real-time corrections, with which the frequency difference between Yb1 and Yb2 is determined and the clock instability is evaluated. FNC, fibre noise canceller; ULE, ultra-low-expansion; PMF, polarization maintaining fibre; GP, Glan polarizer; FS, fibre splitter.

Figure 1 shows a schematic diagram of the experimental setup. Two optical clocks (Yb1 and Yb2) are based on the clock transition ^1^*S*_0_-^3^*P*_0_ of neutral ^171^Yb atoms in an optical lattice. In each system, the one-dimensional (1D) optical lattice is oriented at 54.8° with respect to gravity and is realized by retroreflecting a focused laser beam. The lattice beams, generated from their respective Ti: sapphire lasers LL1 and LL2, are operated at the magic wavelength of 759 nm. LL2 is frequency stabilized to an ultra-low-expansion (ULE) optical cavity with a medium finesse of ~2,000 and its frequency is monitored by a commercial wavemeter with an accuracy of 10 MHz. The laser linewidth is measured to less than 300 kHz and the long-term drift rate is ~75 Hz/s. LL1 is offset locked to LL2, which permits their laser frequency difference to range from sub-MHz to several GHz. After preparing ultracold ytterbium atoms in two successive stages of magneto-optic traps (see Methods), about 1% of the atoms are loaded into the lattice traps for Yb1 and Yb2.

The cold atoms in Yb1 and Yb2 are probed independently by the 578-nm laser pulses. We have prepared two clock lasers for the two systems (see Methods). In each system, an acousto-optic modulator (AOM) operating at around 80 MHz bridges the detuning between the clock laser and the atomic transition and serves as the actuator for feedback control. The clock laser propagates along the lattice trap axis and its linear polarization is purified by the same high-extinction-ratio (10^5^:1) Glan polarizer as used for the lattice laser. After leaving the fibre output end, the 578-nm light need propagate about three-meter free space until it interacts with the atoms. This uncompensated optical path is used in systems of the laser power stabilization and the feedback AOM. The beam waist of the clock laser is three times larger than that of the lattice laser. Two external cavity diode lasers at 649 nm and 770 nm are frequency stabilized on the cavity shared by the LL2 stabilization. Each laser is split via a one-by-two fibre splitter and then is sent to each lattice system.

### Clock-transition spectroscopy and clock operation

**Figure 2:**
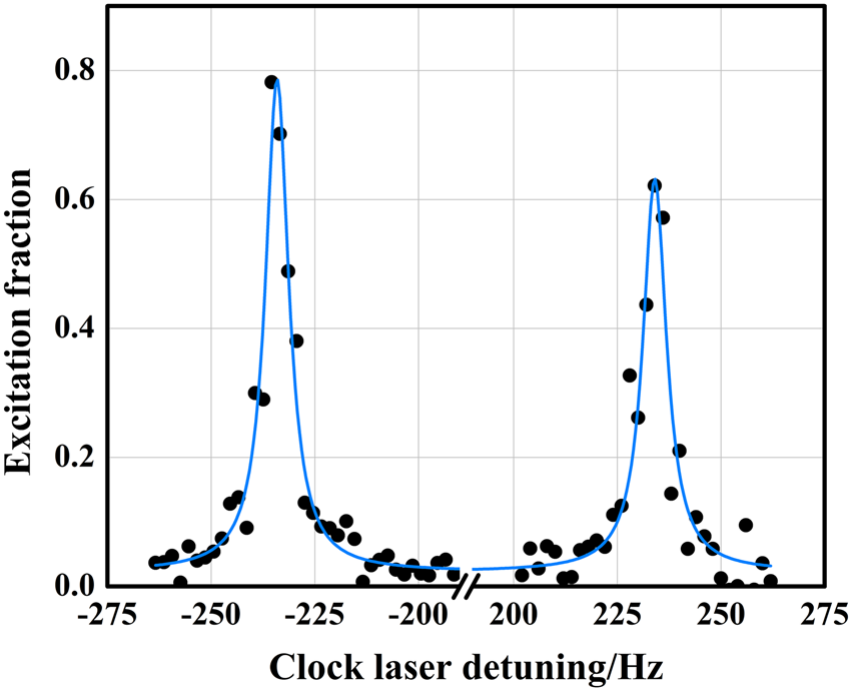
Normalized clock-transition spectra of two ${\boldsymbol{\pi }}$ components. After spin polarization, atoms are probed with a 150-ms-long ${\boldsymbol{\pi }}$-pulse clock laser in the presence of a bias magnetic field of *B*_bias_ = 0.115 mT. Black dots denote experimental data, each being a single measurement requiring 1.335 s. The blue solid line represents a fit with a double-peak Lorentzian function.

By pumping with a proper circularly polarized 556-nm laser pulse, the atoms in the lattice are spin polarized to the Zeeman-sublevel state ^1^*S*_0_, *m*_*F*_ =  +1/2 or ^1^*S*_0_, *m*_*F*_ = −1/2 (see Methods). A bias magnetic field ${B}_{{\rm{bias}}}$ is applied perpendicular to the lattice beam axis (Fig. 1). Before starting clock interrogations, a 10-ms delay allows any transient magnetic fields to decay to background levels. Rabi spectroscopy is performed on the ${\rm{\pi }}$ components of the clock transition by a ${\rm{\pi }}$ pulse of the clock laser. Excitation fraction is extracted from the laser-induced fluorescence and a normalization scheme is employed with the 649-nm and 770-nm lasers. In this way, the shot-to-shot atom-number fluctuation is significantly suppressed. The timescale involved in one cooling-interrogation-detection cycle is 1.335 s. Unfortunately, the observation of the clock-transition spectrum is destructive so that it requires repeated cycles. Figure 2 shows typical normalized clock-transition spectra of two ${\rm{\pi }}$ components with the polarized atoms in the ^1^*S*_0_, *m*_*F*_ = +1/2 and ^1^*S*_0_, *m*_*F*_ = −1/2 state, respectively. The observed excitation fraction of the right ${\rm{\pi }}$ component is only 0.6, which is limited by the scanning resolution of 2 Hz. The obtained spectra have linewidths of 8 Hz, which nearly approaches the Fourier limit by a 150-ms-long clock laser pulse. Taking into account the drift of the clock laser during the two separate scans, the central frequencies of the two spectral lines are separated by 468 Hz when ${B}_{{\rm{bias}}}$ is 0.115 mT.

During the clock operation, the average frequency of the two spectral peaks (Fig. 2) is used to steer the clock laser frequency. To this end, we apply a standard four-point locking procedure. The clock laser alternately interrogates the two ${\rm{\pi }}$ components of the clock transition at each half-maximum point. That is, four interrogation frequencies are separately set at a detuning of ±4 Hz from the peaks. Respective error signals ${\delta }_{1}$ and ${\delta }_{2}$ are derived from the excitation fraction differences for each ${\rm{\pi }}$ component in two independent digital servos. The mean of ${\delta }_{1}$ and ${\delta }_{2}$ (i.e., common-mode signal) provides the frequency correction ${\rm{\Delta }}$ applied to the bridging AOM, thereby completing in principle the feedback control of the clock laser. From the differential-mode error signal $({\delta }_{1}-{\delta }_{2})/2$, the Zeeman splittings are also tracked to monitor the stray magnetic field fluctuation. Taking into account this error in the feedback servo, the sensitivity of the four-point locking is guaranteed and the fluctuation of the first-order Zeeman splitting is rejected. To further improve the locking robustness, a real-time feedforward by fitting the updating ${\rm{\Delta }}$ is applied to the AOM, which compensates the drift of the clock laser.

### Frequency instability by synchronous comparison

**Figure 3:**
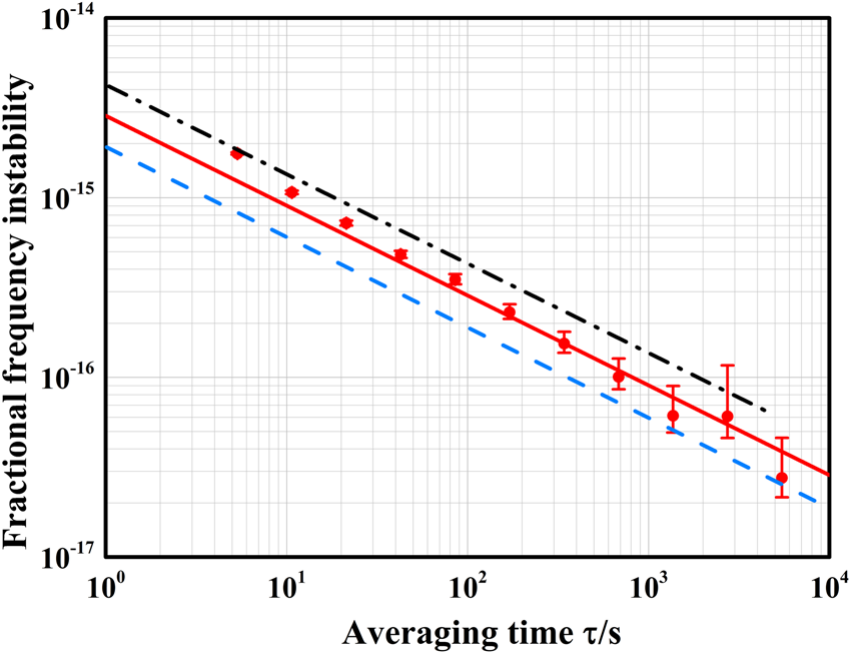
Single clock instability measured with synchronous comparison. The frequency difference $({{\rm{\Delta }}}_{1}-{{\rm{\Delta }}}_{2})/\sqrt{2}$ is used to extrapolate the fractional instability of Yb1 and Yb2, where ${{\rm{\Delta }}}_{1}$ (${{\rm{\Delta }}}_{2}$) is the frequency correction on AOM1 (AOM2). Red points indicate the Allan standard deviation calculated from over 6 hours of data. Error bars indicate $1{\rm{\sigma }}$ uncertainties, assuming a white frequency noise process. The red solid line is a linear fit of the long-term averaging time. The black dash-dot line is the clock instability evaluated by independently locking to the two Zeeman components, which simply represents the instability by the interleaved measurements. The blue dashed line shows the estimated instability from the Dick effect.

As noted above, the optical clocks operate in the periodic-pulsed mode. Only 11% of the cycle time is spent on the clock-transition interrogation. The resulting dead time causes the aliasing of the high frequency noise in the clock laser (i.e., the Dick effect), thereby augmenting the clock instability^28^. Synchronous frequency comparison between optical clocks provides a powerful means to cancel out the laser frequency noise. When comparing the two lattice systems, the interrogation of the two samples of cold atoms in Yb1 and Yb2 are precisely synchronized. The frequency corrections applied to AOM1 and AOM2 are recorded for post-processing to derive the frequency differences and the clock instability (Fig. 1). The fractional frequency instability of a ^171^Yb optical lattice clock given by the Allan standard deviation (Fig. 3) follows the dependence 2.9 × 10^−15^/$\sqrt{\tau }$ for a long-term period (red solid line), and it falls below 4 × 10^−17^ after a 5,000-s averaging.

Instead of the self-comparison from interleaved measurements, we use a slight trick to confirm the benefit from a synchronous comparison. As mentioned in the clock operation, the two virtual clock lasers are alternately stabilized to the two Zeeman components, corresponding to the transition frequencies ${f}_{\pm 1/2}$. When the two Zeeman components are regarded as independent optical clocks, self-comparison can be accomplished just by comparing ${f}_{+1/2}$ and ${f}_{-1/2}$. During the entire locking period, no significant fluctuation of the magnetic field is detected. Judging from the interleaved measurements (black dash-dot line), we find the synchronous comparison yields a 3/2-fold improvement in the clock stability. However, the single clock stability still lies above the estimation from the Dick effect (blue dashed line). This may be attributed to an incomplete rejection of the clock laser noise.

### Systematic frequency shifts

The clock transition is affected by various systematic effects that need to be corrected. We have evaluated the main contributions to the frequency shifts in Yb1 by synchronous comparison with Yb2. Table 1 gives the systematic frequency shifts with their associated fractional uncertainties.

**Table 1:** Systematic frequency shifts for typical experimental conditions and their associated fractional uncertainties in a ^171^Yb optical clock.

Effect	Shift/mHz	Uncertainty/10^−17^
Lattice polarizability	453	15.8
Nonlinear lattice	−97	0.6
Clock laser Stark	0.8	0.6
Density	−570	4.9
Blackbody radiation	−1,289	1.3
First-order Zeeman	0.004	0.2
Second-order Zeeman	−89	4.8
Servo error	0.2	1.0
Total	−1,591.0	17.3

**Figure 4:**
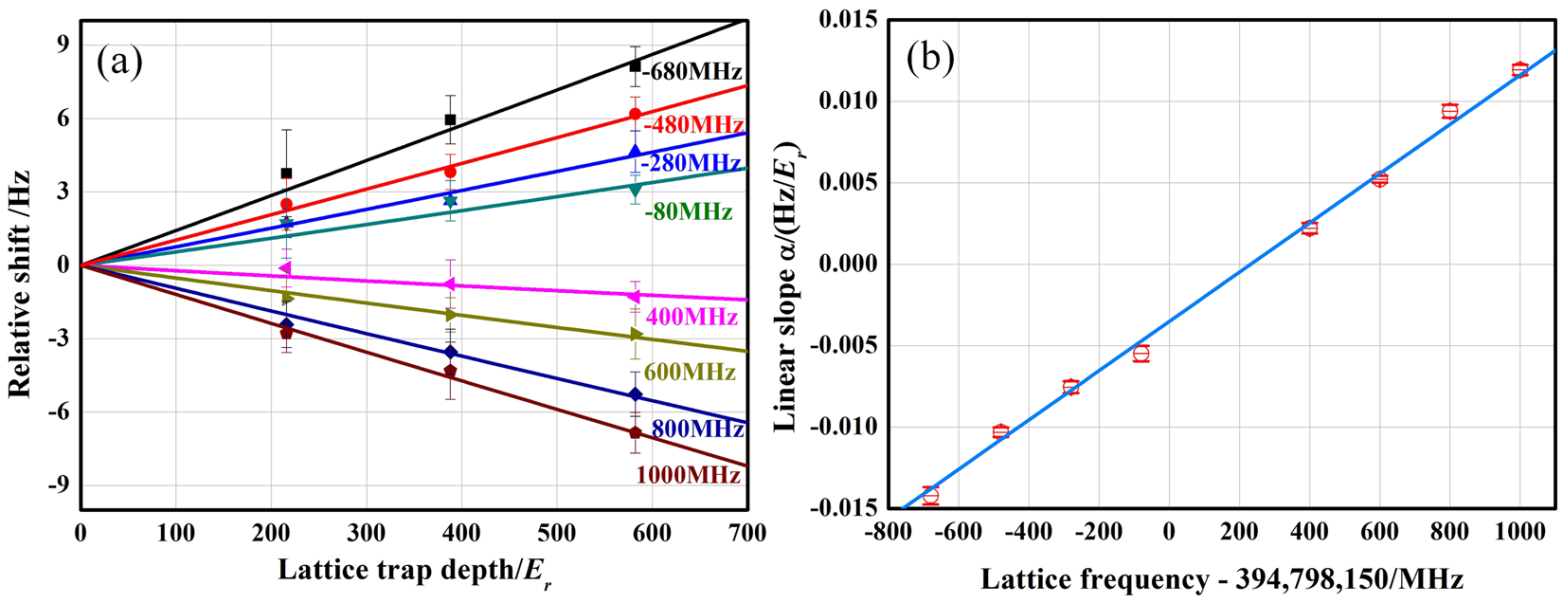
Lattice light shifts and magic frequency determination. (**a**) Relative clock shifts as a function of the lattice trap depth at different lattice frequencies. The solid lines are quadratic fits of the experimental data (coloured points) using equation (1). The labelled frequencies are all relative to 394,798,150 MHz. **(b)** The linear slopes $\alpha$ fitted from (**a**) are plotted as a function of lattice frequency.

Trapping atoms in an optical lattice allows for the cancellation of the Doppler and recoil frequency shifts. However, it comes at the price of inducing an ac Stark shift in the clock transition. The dominant effect comes from the electric dipole polarizability, giving a shift that scales linearly with the trap depth. This shift can be eliminated by tuning the lattice to the magic frequency where the electric dipole polarizabilities of the two clock states are equal^31^. Higher-order couplings, including multi-polarizabilities and hyper-polarizability, prevent a complete cancellation of the light shifts, thus introducing shifts that scale nonlinearly with trap depth. Considering the linear scaling of the atomic temperature with trap depth, the lattice light induced shift can be written as^32^

1 $${\rm{\delta }}{\nu }_{{\rm{clock}}}=-\,\alpha U-\beta {U}^{2},$$

where *U* is the trap depth in units of the lattice recoil energy ${E}_{r}$, and $\alpha$ and $\beta$ are coefficients independent of *U*. We measure the lattice-depth-dependent shifts at different lattice frequencies from the synchronous comparison, as shown in Fig. 4(a). The experimental conditions are changed only in the Yb1 system. The frequency of LL1 can be varied easily by tuning the rf reference in the offset locking system (Fig. 1). Each group of data is fitted using equation (1) to give a linear slope $\alpha$ by fixing $\beta$ at −2.85(10) × 10^−7^ Hz/*E*_*r*_^2^ (ref.^32^). The slope of the fitting lines is plotted as a function of the lattice frequency, as shown in Fig. 4(b). Finally, the slope of $\alpha$ is determined to be 1.51(2) × 10^−5^ Hz/(MHz *E*_r_) by a linear fitting and the magic frequency is found at 394,798,381.5(9.3) MHz. Considering the typical operating conditions for the lattice laser are $582(6){E}_{r}$ and 394,798,330 MHz, the lattice polarizability shift is estimated to be 0.453(82) Hz, which corresponds to a fractional uncertainty of 1.58 × 10^−16^. From the fixed $\beta$, the nonlinear shift is −0.097(3) Hz with an uncertainty of 6 × 10^−18^.

The clock laser intensity required for Rabi spectroscopy scales as the inverse of the square of the Rabi time^26^. For longer interrogation times, the frequency shift decreases significantly. We calculate this shift using values reported in ref.^27^. By rescaling to the Rabi time of 150 ms, the light shift of the clock laser is 0.8 mHz with an uncertainty of 6 × 10^−18^.

**Figure 5:**
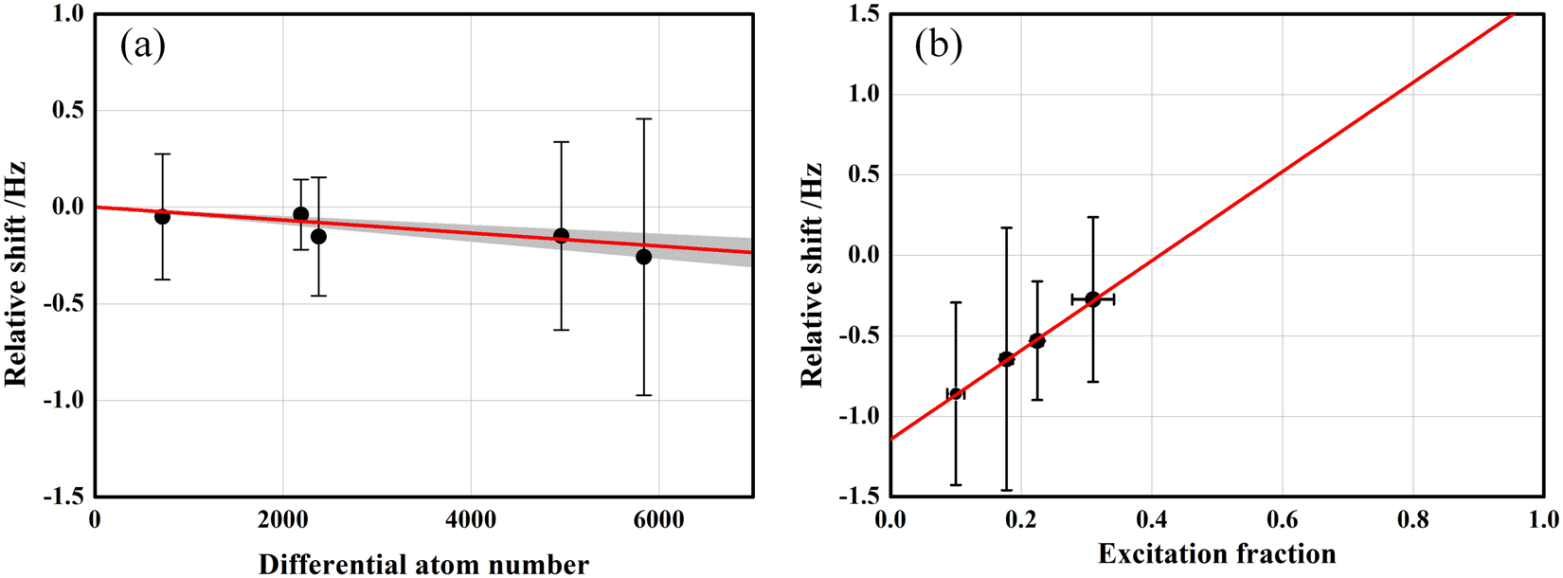
Measurement of the density shift. (**a)** Measured density shift by changing the atom number when excitation fraction is 0.22(1). Experimental data (black dots) are shown with a linear fit (red solid line). The grey shaded area represents the statistical uncertainty of the fit. **(b)** Measured density shift by changing the excitation fraction with atom number of 5,840(260).

The density shift is in general described by different partial-wave scatterings. Despite the employment of ultracold spin-polarized fermions, the *s*-wave scattering may arise from various inhomogeneous Rabi excitations^33,34^. In a ^171^Yb optical lattice clock, the density shift has been demonstrated to be dominated by *p*-wave cold collisions^35^. In this study, we carefully avoid any inhomogeneous Rabi excitations and investigate the density-induced frequency shift by decreasing the atom number in Yb1 while that in Yb2 remains constant. The atom number is reduced during first-stage cooling by a variation of the laser power for the Zeeman slower, because this operation does not change the lattice trapping conditions. We have reduced the atom number by nearly a factor of eight from the typical experimental conditions. The density shift as a function of the differential atom number is shown in Fig. 5(a). As this shift is also sensitive to the excitation fraction, we also measure this dependence as well, as shown in Fig. 5(b). Under typical experimental conditions, the atom number is calibrated at 5,840(260) and the excitation fraction during the clock operation is 0.22(1). Thus, the density shift is estimated to be −0.570(25) Hz.

Blackbody radiation (BBR) from the finite-temperature surroundings shifts the clock transition. The temperature dependence of the clock BBR shift is given by^36^

2 $${\rm{\Delta }}{\nu }_{{\rm{BBR}}}=-\frac{1}{2h}{\langle {E}^{2}\rangle }_{{T}}{\rm{\Delta }}\alpha (0)[1+{{\rm{\eta }}}_{{\rm{clock}}}(T)],$$

where *h* is the Planck constant, ${{E}^{2}}_{T}\approx {(831.9V/{\rm{m}})}^{2}{(T/300{\rm{K}})}^{4}$ the mean-squared electric field inside the blackbody at absolute temperature *T*, ${\eta }_{{\rm{clock}}}$ the dynamic correction, and ${\rm{\Delta }}\alpha (0)$ the differential static polarizability of clock states. For ^171^Yb, ${\rm{\Delta }}\alpha (0)$ has been measured with high accuracy and $\eta$ has also been well understood^37,38^. To evaluate the BBR shift, complete knowledge of the temperature around the atoms becomes an important issue. In this study, temperatures on the outer surface of the science chamber were measured during the clock operation period using seven calibrated platinum resistance thermometers. The temperature gradients were numerically simulated by a finite-element radiation analysis on the chamber structure in accordance with the measured temperature data^39^. We obtained the temperature distribution inside the chamber and around the cold ytterbium atoms. The BBR shift is estimated to be −1.289(7) Hz with an uncertainty of 1.25 × 10^−17^.

**Figure 6:**
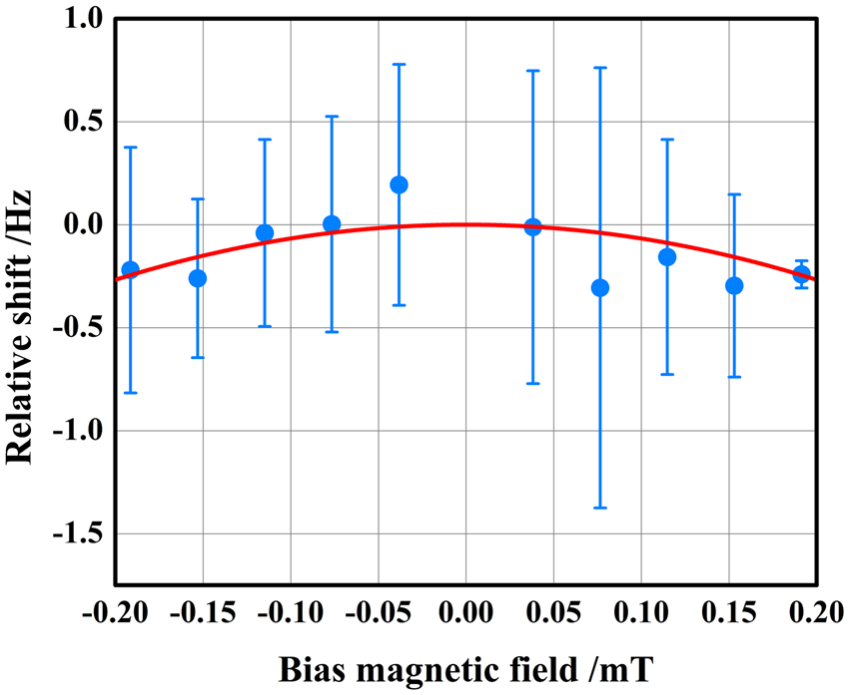
Measurement of the second-order Zeeman shift. Experimental data (blue dots) are fitted with quadratic function *aB*^2^ for which *a* is −6.7(1.8) MHz/T^2^.

In general, the first-order Zeeman shift and the lattice vector shift are cancelled out by averaging the central frequencies of the two ${\rm{\pi }}$ components of the clock transition. However, if the background magnetic field drifts appreciably between measurements, a residual first-order Zeeman shift occurs. Using a three-dimensional digital magnetometer to monitor the stray magnetic field near the science chamber, we observe a background linear drift of 0.002(9) ${\rm{\mu }}T$ per hour. By interpolating the drift data to the Zeeman splitting, the residual first-order Zeeman shift is estimated to be 0.004 mHz with an uncertainty of 2 × 10^−18^. The second-order Zeeman shift arising from a bias magnetic field was also investigated. The resulting data (Fig. 6) are fitted with a *aB*^2^ function to determine the second-order sensitivity. The second-order Zeeman effect is predicted to have dependence [−6.7(1.8) MHz/T^2^]*B*^2^, where *B* is the bias field magnitude. The second-order coefficient is in good agreement with previous work^27,40^. When *B* is 0.1148(3) mT, the second-order Zeeman shift is estimated to be −89(25) mHz.

Servo error is evaluated by simply calculating the mean in-loop error signal $({\delta }_{1}+{\delta }_{2})/2$ and the frequency instability is determined from the Allan standard deviation. The fractional frequency shift is estimated to be 0.3 × 10^−18^.

## Discussion

By synchronous interrogation of two lattice systems, we have characterized a ^171^Yb optical clock in terms of frequency instability and systematic uncertainty. Although the short-term stability in the synchronous measurement has an improvement by judging from the interleaved measurements, it is still worse than the estimation from the Dick effect. As each clock laser beam propagates in free space for a long distance to probe the atoms after the fibre transmission, the instability degradation can be attributed to additional noises from these uncompensated free-space optical paths. The systematic uncertainty is mainly limited by the lattice polarizability shift (see Table 1). We expect that the lattice contributions can be reduced to the 10^−17^ level once the instability-degradation issue is solved. Other frequency shifts can also be controlled with higher precision. For example, the Zeeman shift may be reduced by applying a smaller bias magnetic field. Moving atoms into a cryogenic chamber is an effective method for reducing the BBR shift. In addition, systematic evaluation of Yb2 is ongoing.

## Methods

### Preparation of cold atom samples

As a detailed description of the cooling and trapping system is presented elsewhere^30,41–43^, a brief description is given here. Initially, thermal ytterbium atoms effuse from the heated oven followed by two collimators with a separation of 12 cm and diameters of 3 and 6 mm. They are transversally cooled in a two-dimensional optical molasses, and then decelerated longitudinally in a Zeeman slower operated with light red-detuned by 720 MHz from the ^1^*S*_0_-^1^*P*_1_ cycling transition at 399 nm. The cold atom sample is prepared in two successive magneto-optical traps (MOTs). As many as 10^7^ atoms are captured in the 399-nm MOT with a temperature of 1 mK. Further cooling is performed with the 556-nm MOT based on the ^1^*S*_0_-^3^*P*_1_ transition, yielding 10^6^ atoms at a temperature of $10\,{\rm{\mu }}K$. In the centre of the science chamber, cold atoms are overlapped with a tilted one-dimensional (1D) optical lattice.

### Spin polarization

Initially, atoms in the optical lattice are equally distributed in two Zeeman sublevels of the hyperfine ^1^*S*_0_, *F* = 1/2 state. Spin polarization is performed by optical pumping to either Zeeman sublevels. The pumping laser at 556 nm with circular polarization is chosen to be near resonant with the ^1^*S*_0_, *F* = 1/2-^3^*P*_1_, *F* = 3/2 transition, where a bias magnetic field ${B}_{{\rm{bias}}}$ splits the adjacent ${\rm{\pi }}$ components by 3.45 MHz. Alternating between the ${\sigma }^{+}$ and ${\sigma }^{-}$ polarization of the pumping laser is realized by reversing the ${B}_{{\rm{bias}}}$ direction. For the ${\sigma }^{+}$ polarized beam, atoms are excited from the ^1^*S*_0_, *m*_*F*_ = −1/2 state to the ^3^*P*_1_, *m*_*F*_ = +1/2 state. Some atoms that decay on a ${\sigma }^{+}$-transition path are pumped again, whereas others that decay on a ${\rm{\pi }}$-transition path accumulate in the ^1^*S*_0_, *m*_*F*_ = +1/2 dark state as the upper state is resolvable for the pumping laser. Similarly, the ${\sigma }^{-}$ polarized beam pumps atoms into the ^1^*S*_0_, *m*_*F*_ = −1/2 state.

### Ultrastable clock lasers

Two ultrastable clock lasers at 578 nm were prepared for the Yb1 and Yb2 systems. They are both generated by sum frequency mixing, each from a 1319-nm Nd:YAG laser with a 1030-nm fibre laser in a periodically poled lithium niobate (PPLN) waveguide. The clock laser CL1 in Yb1 is frequency stabilized to a thermal-noise-limited ULE cavity with finesse of ~6.7 × 10^5^. The laser linewidth is reduced to ~1 Hz and it exhibits a fractional frequency instability of 1.3 × 10^−15^ at 1 s averaging time^44,45^. We implement a tight phase locking between CL2 and cavity-stabilized CL1 (Fig. 1). Therefore, the two clock lasers are essentially subjected to the same noise. Each clock laser is delivered to the optical lattice through a 15-m long polarization maintaining optical fibre. Fibre length fluctuations are actively compensated by installing a fibre noise canceller^46^.

### Data availability

The data that support the findings of this study are available from the corresponding authors on reasonable request.
